# Supplementary material for: Bovine C-X-C Motif Chemokine Ligand 14 Expression Is Regulated by Alternative Polyadenylation and MicroRNAs
Source: Animals (Basel). 2023 Sep 30;13(19):3075. doi: 10.3390/ani13193075 (PMC10571712; doi:10.3390/ani13193075)

Figure S1. Western Original Image

Full original blots used for Figure 3C. Each blot membrane was cut based on the standard band positions and then incubated with the appropriate antibodies. The bands in the article are marked by red box. The results of other repeated experiments are marked with blue boxes.

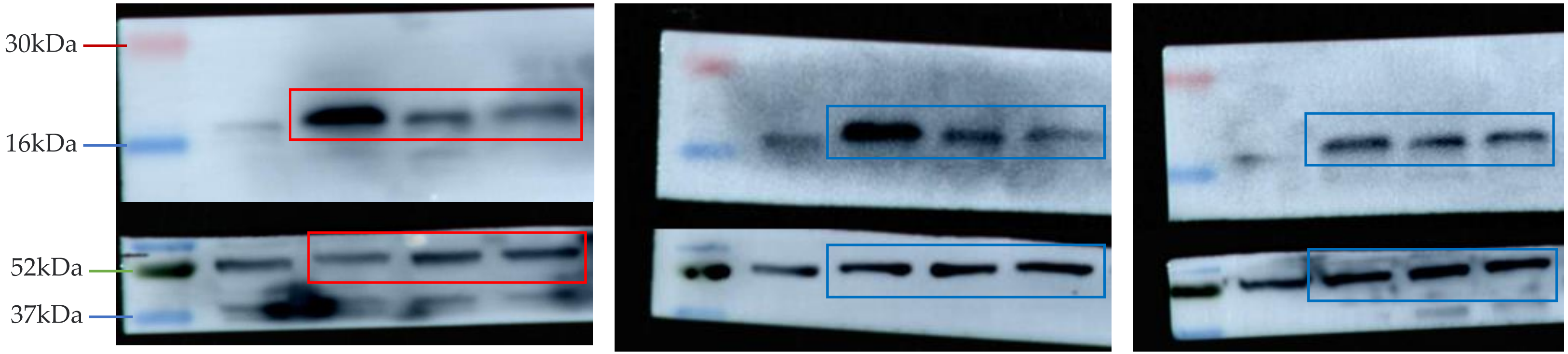

Full original blots used for Figure 5B. Each blot membrane was cut based on the standard band positions and then incubated with the appropriate antibodies. The bands in the article are marked by red box. The results of other repeated experiments are marked with blue boxes.

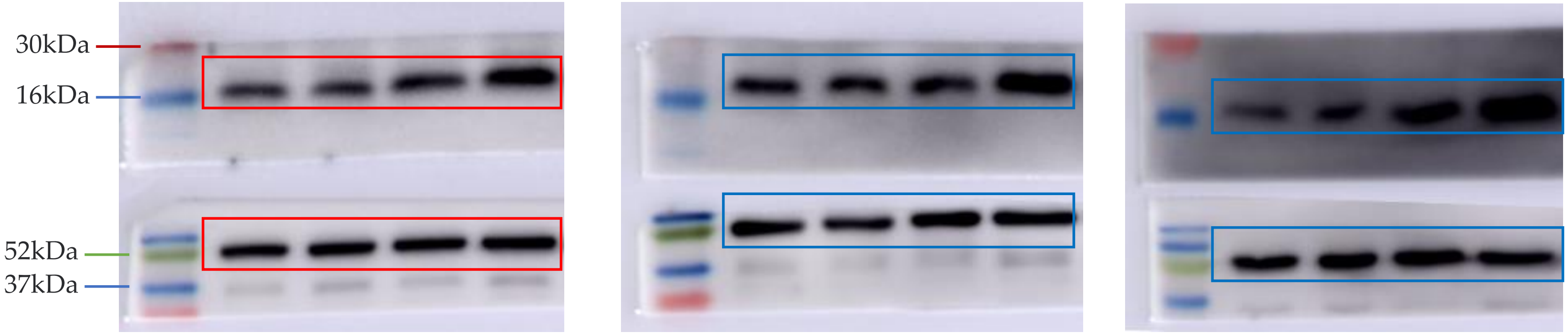

Full original blots used for Figure 5D. Each blot membrane was cut based on the standard band positions and then incubated with the appropriate antibodies. The bands in the article are marked by red box. The results of other repeated experiments are marked with blue boxes.

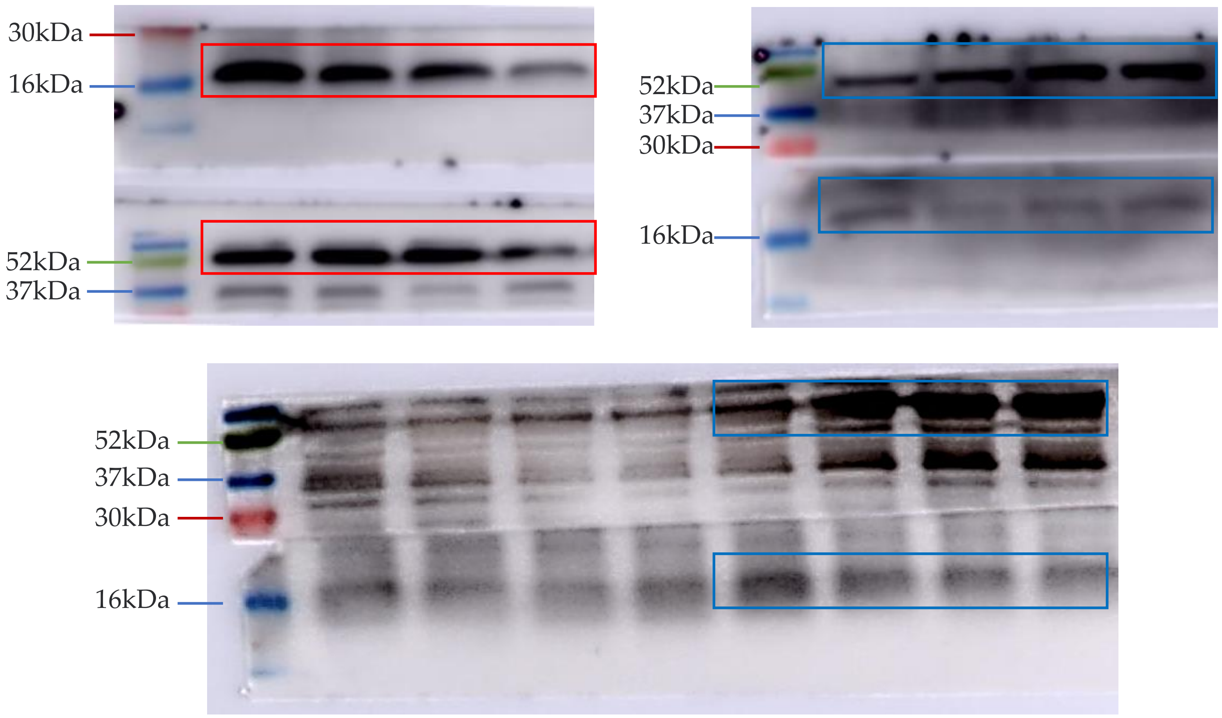

Supplement: Supplementary file 1 [file animals-13-03075-s001.zip › Figure S1.pdf]
