# Supplementary material for: Bovine C-X-C Motif Chemokine Ligand 14 Expression Is Regulated by Alternative Polyadenylation and MicroRNAs
Source: Animals (Basel). 2023 Sep 30;13(19):3075. doi: 10.3390/ani13193075 (PMC10571712; doi:10.3390/ani13193075)
Supplement: Supplementary file 1 [file animals-13-03075-s001.zip › Table S1.pdf]

Table S1. Primers used for 3′ RACE and Construction of recombinant vector

Primer name	Sequence (5′-3′)	Purpose
GSP1	F: AGCGGTTCATCAAATGGTAC	3′ RACE
GSP2	F: TATACAACTACAGACACACGTG	3′ RACE
3′ UTR-S	F: CCGCTCGAGGGTGAAAAATCCCTAAT<br>R: TTGCGGCCGCGCTTTGTAACAATATTTAAT	Construction of vector
3′ UTR-L	F: CCGCTCGAGGGTGAAAAATCCCTAAT<br>R: ATAAGAATGCGGCCGCTACATTTCAAATATAT	
CXCL14-CDS	F: CGACAAGCTTATGCGCATGAGGCTCCTGACCGC<br>R: TAGGGATTTTTCACCCTATTCTTCGTAGACCCTGCG	
CXCL14-S	F: GTCTACGAAGAATAGGGTGAAAAATCCCTAATGGGAAAACT<br>R: TGTCTGGATCCCCGCGCTTTGTAACAATATTTAATTTGTG	
CXCL14-L	F: GTCTACGAAGAATAGGGTGAAAAATCCCTAATGGGAAAACT<br>R: TGTCTGGATCCCCGCACATTTCAAATATATTTTATTACTT	
3′ UTR-LM or CXCL14-LM	F: CACAGGCATAAGACACAATCCAAATATTG<br>R: CAATATTTGGATTGTGTCTTATGCCTGTG	site-specific mutagenesis
3′ UTR-L-Mut17	F: CAAATACACCCTTACCGAGGGTCAGTTTTTACATTT<br>R: CGGTAAGGGTGTATTTGTAACAATATTTAATTTGTGTCTTATGC	
3′ UTR-L-Mut 150	F: GGACCAAACCAATCTTCTCTCTAACATGAGCATTTGTG<br>R: GAAGATTGGTTTGGTCCTTAAGGACCATCTATCTGCTTC	
3′ UTR-L-Mut217	F: GGAATCCATCACACTAAAGCAATCTCTCTGTGATTCTCG<br>R: TTAGTGTGATGGATTCCATTGTCGAGAGGGAAAG	

Note: (1) Except for the 3′ RACE primer, the other primer names represent the forward and reverse primers used in the construction of these recombinants. (2) The underline in the primer pairs for the construction of the 3′ UTR-LM vector indicates the mutation of the "ATTAAA" sequence of PAS1 to "TCCAAA". (3) The underline in the primer pairs for the construction of the 3′ UTR-L-Mut17, 3′ UTR-L-Mut 150, and 3′ UTR-L-Mut217 vector indicates the mutation in the corresponding sequence of binding sites in the seed region of miRNAs.
