# Supplementary material for: Bovine C-X-C Motif Chemokine Ligand 14 Expression Is Regulated by Alternative Polyadenylation and MicroRNAs
Source: Animals (Basel). 2023 Sep 30;13(19):3075. doi: 10.3390/ani13193075 (PMC10571712; doi:10.3390/ani13193075)
Supplement: Supplementary file 1 [file animals-13-03075-s001.zip › Table S2.pdf]

Table S2. List of miRNAs

miRNAs name	Sequence (5′-3′)	
miR-17-5p mimics	S: CAAAGUGCUUACAGUGCAGGUAGU	AS: UACCUGCACUGUAAGCACUUUGUU
miR-150 mimics	S: UCUCCCAACCCUUGUACCAGUGU	AS: ACUGGUACAAGGGUUGGGAGAUU
miR-217 mimics	S: UACUGCAUCAGGAACUGAUUGGAU	AS: CCAAUCAGUUCCUGAUGCAGUAUUA
miR-671 mimics	S: AGGAAGCCCUGGAGGGGCUGGAG	AS: CCAGCCCCUCCAGGGCUUCCUUU
miR-874 mimics	S: CUGCCCUGGCCCGAGGGACCGA	AS: GGUCCCUCGGGCCAGGGCAGUU
Negative control	S: UUCUCCGAACGUGUCACGUTT	AS: ACGUGACACGUUCGGAGAATT

Note: S and A denote the sense chain and the anti-sense chain, respectively.
