# Supplementary material for: Bovine C-X-C Motif Chemokine Ligand 14 Expression Is Regulated by Alternative Polyadenylation and MicroRNAs
Source: Animals (Basel). 2023 Sep 30;13(19):3075. doi: 10.3390/ani13193075 (PMC10571712; doi:10.3390/ani13193075)
Supplement: Supplementary file 1 [file animals-13-03075-s001.zip › Table S3.pdf]

Table S3. Primers used for real-time quantitative PCR

Primer name	Sequence (5′-3′)	
bCXCL14	F: ACTGCGAGGAGAAGATGGTT	R: CTGTGAGAAAGAAAGGGTTTGT
mCyclinD1	F: GCGTACCCTGACACCAATC	R: CCTCCTCTTCGCACTTCTG
mCyclinE	F: GCCTCGGAAAATCAGACCA	R: CCATCAGCCAATCCAGAAGAAC
mCullin3	F: CGGGATATTGGCCTACTCA	R: GACCATAAAAGCTGGCATTG
mGAPDH	F：CGGGGTCCCAGCTTAGGTTC	R：GCCCAATACGGCCAAATCCGT
hGAPDH	F: ACAACTTTGGTATCGTGGAAGG	R: GCCATCACGCCACAGTTTC

Note: b, bovine; m, mouse; h, human.
